# Jaundice in Secondary Syphilis

**Published:** 1911-02-25

**Authors:** 


### Medicine.

### JAUNDICE IN SECONDARY SYPHILIS.

Sometimes the results of secondary syphilitic hepatitis are very obscure, and are associated with severe intra-abdominal pain and with pyrexia and general ill-health. Laparotomy has more than once been resorted to on their account under the belief that the symptoms pointed to leakage from a gastric or duodenal ulcer or an abscess in connection with the liver. That jaundice should sometimes result from secondary syphilitic inflammation in the liver is not surprising, therefore, and sometimes the changes in the bile pigments are such that, although they produce yellow discolouration of the tissues, they do not give the ordinary tests for bile pigments in the urine—in other words, so-called "acholuric" jaundice. The following is an instance in point:—

A young woman, aged nineteen, came into hospital for jaundice and an eruption upon the skin. The latter was a syphilitic roseola, almost confluent in type, and it had been out for a fortnight, although, as is so often the case in women, the patient had had no knowledge of the primary chancre. There was general enlargement of the lymphatic glands, particularly in the inguinal and sub-maxillary regions; but there were no vulval lesions, no sore throat, no headache, and no fever. The jaundice had appeared a week previous to the roseola, beginning gradually, attaining its maximum in three or four days, and persisting from that time without variation. There had been no previous jaundice and no noteworthy gastro-intestinal trouble, nor was there any pain to suggest a gall-stone.

The urine was not very highly coloured, and did not give the Gmelin's reaction. The stools were not decolourised, and there was no constipation. The liver dulness was neither increased nor diminished, and the liver itself could not be felt, but the spleen was increased in size. The pulse-rate was eighty, and there were no cardiac or pulmonary abnormalities. There was no itching. Treatment by intra-muscular injections of the biniodide of mercury in a watery solution was adopted, and the jaundice steadily diminished. Numerous blood examinations were made; the most important point determined thereby was that the blood serum contained bile pigments, although the urine did not. There was only slight anæmia, whilst the leucocytes averaged from 10,000 to 30,000 per cubic millimetre.

The case was clearly one of syphilitic acholuric jaundice of the variety sometimes attributed to "roseola of the biliary passages." The diagnosis in the present instance was easy enough on account of the copious eruption upon the skin, but in other instances the secondary syphilitic nature of hepatic symptoms may not be equally clear, though it is important to bear their possibility in mind.

